# Psychological coherence, inclusive leadership and implicit absenteeism in obstetrics and gynecology nurses: a multi-site survey

**DOI:** 10.1186/s12888-022-04137-1

**Published:** 2022-08-03

**Authors:** Yu Jin, Qingquan Bi, Guiqi Song, Jun Wu, Hui Ding

**Affiliations:** 1 Hefei, China; 2 Binhu Healthcare Center, The First Affiliated Hospital of USTC, Division of Life Sciences and Medicine, University of Science and Technology of China, Hefei, Anhui 230001 China; 3 Department of Education, The First Affiliated Hospital of USTC, Division of Life Sciences and Medicine, University of Science and Technology of China, Hefei, Anhui 230001 China; 4 Department of Geriatrics, The First Affiliated Hospital of USTC, Division of Life Sciences and Medicine, University of Science and Technology of China, Hefei, Anhui 230001 China

**Keywords:** Psychological coherence, Inclusive leadership, Absenteeism, Nurse, Care, Management

## Abstract

**Background:**

Implicit absenteeism is very common among clinical nurses. We aimed to evaluate the role of psychological coherence in the inclusive leadership and implicit absenteeism among obstetrics and gynecology nurses, to provide evidence to the clinical management of nurses.

**Methods:**

Through the convenience sampling method, a survey of gynecology nurses in tertiary hospitals in 16 cities of Anhui Province, China was conducted using the General Information Questionnaire, the Stanford Implicit Absence Scale, the Inclusive Leadership Scale and the Sense of Coherence Scale. Statistical analysis was performed by SPSS 20.

**Results:**

A total of 1080 nurses were included with an effective response rate of 93.5%. The average score of nurses' recessive absenteeism in this study was (16.8 ± 0.15). The average of inclusive leadership score was (34.25 ± 7.23). The average score of psychological coherence score of obstetrics and gynecology nurses was (55.79 ± 8.28). Pearson correlation analysis showed that there was a relationship between implicit absenteeism behavior, inclusive leadership, and the level of psychological coherence in obstetrics and gynecology nurses (all *P* < 0.05). Linear regression analysis indicated that psychological coherence played a partial mediating role between inclusive leadership and obstetrics and gynecology nurses' implicit absenteeism (all *P* < 0.05).

**Conclusions:**

Obstetrics and gynecology nurses have serious recessive absenteeism with low sense of psychological coherence and inclusive leadership. Nursing managers should improve the psychological coherence through effective interventions, thereby reducing the incidence of implicit absenteeism.

## Background

Implicit absenteeism refers to employees who are on the job, but due to physical or mental health problems, their lack of work engagement, low work efficiency, and a behavioral state of "people are distracted" [1, 2]. In recent years, with the enrichment of nursing connotation and the continuous increase of people's demands for health, the psychological pressure on nurses has been increasing [3]. The Chinese national mental health report [4] has indicated that the incidence of hidden absenteeism in nurse population is 3 to 4 times higher than that of ordinary enterprise employees. Therefore, the prevention and intervention of nurses' implicit absenteeism is of great significance to nurses' mental health and quality of life [5].

Psychological coherence is a core concept of the Beneficial Health Model and Stress Cognitive Style, it refers to an individual's ability to effectively manage and utilize internal and external resources when confronted with stressors in life, and to resolve tensions in a way that maintains health [6]. Psychological coherence expresses an individual's general, lasting, dynamic sense of self-confidence [7]. Previous studies [8, 9] have shown that psychological coherence is the key to effectively cope with stressful stimuli, maintain physical and mental health and avoid physical and psychological problems. A high level of psychological coherence is negatively correlated with negative emotions such as anxiety and depression, and is positively associated with high-quality health status [10, 11]. At present, many scholars in China have carried out researches on implicit absenteeism or psychological sense of coherence, but few studies have been carried out on the correlation between the two among nurses.

Some studies [12, 13] have pointed out that the leadership style perceived by obstetrics and gynecology nurses in the work environment will affect their level of psychological coherence to a certain extent, and a lower psychological coherence will reduce the enthusiasm and commitment of nurses at work, and increase incidence of hidden absenteeism. To this end, we come to the hypothesis that psychological coherence may mediates the inclusive leadership and implicit absenteeism among obstetrics and gynecology nurses. Therefore, we aimed to explore the relationship between the level of psychological coherence, implicit absence behavior and inclusive leadership among obstetrics and gynecology nurses, to clarify the mechanism of action among them, thereby providing evidence support for improving the management of obstetrics and gynecology nurses.

## Methods

### Ethics

In this study, all methods were performed in accordance with the relevant guidelines and regulations. This present study had been approved by the ethical committee of Anhui Provincial Hospital (approval number:2021KY-277). And written informed consent had been obtained from all the included participants.

### Study population

This study used the convenience sampling method to conduct a questionnaire survey from October to December 2020. Obstetrics and gynecology nurses in tertiary hospitals of 16 cities in Anhui Province, China were selected. The inclusion criteria for surveyed nurses were: ①Clinical on-the-job registered nurses; ②The nurse was working in the department of obstetrics and gynecology; ③Nurses gave informed consent to this study and participated voluntarily. Exclusion criteria were as following: ① training nurses or rotating nurses were excluded; ②nurses who did not agree to participate in this survey.

### Survey tools

#### Questionnaire for general information

We designed the Questionnaire for General Information for collecting the characteristics of surveyed nurses, including gender, age, professional title, educational background, working years, marital status, whether there are children, employment relationship, the number of night shifts per month, and the number of patients in charge of day and night shifts.

#### Stanford hidden absence scale

The scale was compiled by Koopman et al. [14] and translated and revised by Chinese scholar Zhao Fang [15]. The Cronbach’s alpha of translated Stanford Hidden Absence Scale is 0.79 with good reliability and validity. The scale is used to estimate the productivity loss caused by the implicit absence behavior caused by a specific health condition in the past month, and it includes a total of 6 items. Each item adopts a “5-point” Likert scale method, ranging from "completely disagree" to "completely agree". The score for each item ranges from 1 to 5, and the total score ranges from 6 to 30. The higher the score, the greater the loss of productivity due to health problems and the less effective attendance.

#### Inclusive leadership scale

Inclusive Leadership Scale was compiled by Carmeli et al. [16]. in 2010, and it was translated and revised by Chinese scholar Peng Wei et al. [17]. The Cronbach’s alpha of translated Inclusive Leadership Scale is 0.81 with good reliability and validity. The scale includes 3 dimensions of openness, effectiveness, and amiability, with a total of 9 items. The scale adopts “a 7-point” Likert scale, from "completely inconsistent" to "completely consistent". The score for each item ranges from 1 to 7, and the total score ranges from 9 to 63. The higher the score, the better the inclusive leadership behaviors perceived by nurses.

### Psychological coherence scale

The psychological coherence scale was compiled by Antonovsk [18], it was translated and revised by Bao [19]. The Cronbach’s alpha of translated psychological coherence scale is 0.77 with good reliability and validity. The scale contains the sense of understanding (5 items), sense of control (4 items), sense of meaning (4 items and 3 dimensions, a total of 13 items). The scale adopts a “7-level” scoring method, in which items 1, 2, 3, 8, and 13 are reverse scoring, the score of each dimension is the sum of the scores of the items of the dimension. The total score of the scale is the sum of the scores of each dimension, and the total score range is from 13 to 91 points, with higher scores indicating higher levels of psychological coherence.

#### Data collection

In this study, following the principle of informed consent, an online questionnaire survey was conducted among the respondents. We contacted the head nurses of obstetrics and gynecology departments in tertiary hospitals of 16 cities of Anhui Province, we distributed the online links of questionnaires to them to finish the survey. All the name of the respondents had been anonymized by the web background. Before the survey, the purpose and meaning of the survey, the filling method of each questionnaire, and matters needing attention were explained to the nurses. In order to ensure the quality of the questionnaire, questions such as age and working years in the questionnaire have been set with a maximum number of characters. All answers must be filled out before submission. And two authors discreetly check the questionnaires to ensure the integrity and validity of the questionnaires.

### Statistical methods

SPSS 20.0 software was used to analyze the collected data. We analyzed and presented the demographic characteristics of overall population firstly. Measurement data were expressed as (mean ± standard deviation), and independent samples t-test was used for comparison between groups. Enumeration data were expressed as percentage (%), and continuous-corrected chi-square test was used for comparison between groups. Pearson correlation analysis was performed to analyze the correlation of psychological consistency, inclusive leadership, and implicit absence. Linear regression was used to analyze the effects of inclusive leadership and psychological coherence on implicit absence. In addition, we used inclusive leadership as the independent variable and implicit absence as the dependent variable to examine the mediating role of psychological coherence. In this study, *p* < 0.05 was considered statistical significance.

## Results

### The characteristics of surveyed nurses

A total of 1155 questionnaires were distributed and received, 75 questionnaires were excluded from logical errors and omissions, and 1080 questionnaires were finally valid, with an effective response rate of 93.5%. As presented in Table 1, a total of 1080 nurses were included in this study, all of whom were female. Over 85% of the participants were below 40 years of age. In terms of work, 60% of the respondents have less than 5 night shifts per month. Nearly 70% of the respondents were nursing fewer than 16 patients during the day shift. Nearly 60% of the respondents were nursing more than 32 patients in the night shift.

**Table 1:** The general information and characteristics of surveyed nurses (*n* = 1080)

Variables	Cases (n)	Percentage (%)
Gender		
Female	1080	100.0%
Age(y)		
≤ 30	576	53.3%
31 ~ 40	344	31.9%
40 ~ 50	140	13.0%
≥ 50	20	1.8%
Job title		
Nurse	316	29.3%
Nurse Practitioner	436	40.4%
Nurse-in-charge	311	28.8%
Professor of Nursing	17	1.5%
Educational level		
High school	37	3.4%
College	446	41.3%
Bachelor	596	55.2%
Master	1	0.1%
Working experience(y)		
< 1	47	4.3%
1 ~ 5	357	33.1%
6 ~ 10	301	27.9%
≥ 10	375	34.7%
Marital status		
Married	751	69.6%
Unmarried	307	27.9%
Divorced	20	1.9%
Other	2	0.2%
Having child		
Yes	704	65.2%
No	376	34.8%
Employment relationship		
Formal staffing	232	21.5%
Contract	836	77.4%
Dispatch	12	1.1%
Number of night shifts per month		
0 ~ 5	652	60.4%
5 ~ 10	360	33.3%
≥ 10	68	6.3%
Number of patients in day shift nursing		
1 ~ 8	313	29.0%
9 ~ 16	423	39.2%
> 16	344	31.8%
Number of patients in night shift nursing		
1 ~ 16	224	20.7%
17 ~ 32	227	21.0%
> 32	629	58.3%

### Implicit absenteeism of obstetrics and gynecology nurses

The average score of nurses' recessive absenteeism in this study was 16.8 ± 0.15 points, of which the minimum value was 6 points and the maximum value was 30 points. Previous study [20] has reported that more than half of nurses have implicit absenteeism and take the median of nurses' recessive absence total score as the cut-off point to differentiate the implicit absenteeism. Therefore, we took the median of the nurses' recessive absence total score as the cut-off point to divide the nurses into low and high implicit absenteeism groups. There were 593 nurses with high implicit absenteeism (54.91%) and 487 low implicit absenteeism (45.09%). The characteristics of nurses with high implicit absenteeism and low implicit absenteeism were presented in Table 2. There were significant differences in the job title, working experience, employment relationship, number of night shifts per month between nurses with high and low implicit absenteeism (all *P* < 0.05). No significant differences were found in the in the gender, age, educational level, marital status, having child, number of patients in day shift nursing, number of patients in night shift nursing between nurses with high and low implicit absenteeism (all *P* > 0.05).

**Table 2:** The implicit absenteeism of 1080 obstetrics and gynecology nurses

Variables	Low implicit absenteeism group (*n* = 487)		High implicit absenteeism group (*n* = 593)		*χ*^*2*^	*P*
	Cases	Percentage	Cases	Percentage		
Gender						
Female	487	100.0%	593	100.0%		
Age(y)					8.901	0.03
≤ 30	241	49.5%	335	56.5%		
31 ~ 40	158	32.4%	186	31.4%		
40 ~ 50	77	15.8%	63	10.6%		
≥ 50	11	2.3%	9	1.5%		
Job title					22.194	< 0.01
Nurse	152	31.2%	164	27.7%		
Nurse Practitioner	166	34.1%	270	45.5%		
Nurse-in-charge	155	31.8%	156	26.3%		
Professor of Nursing	14	2.9%	3	0.5%		
Educational level					4.848	0.18
High school	13	2.7%	24	4.0%		
College	214	43.9%	232	39.1%		
Bachelor	259	53.2%	337	56.8%		
Master	1	0.2%	0	0.0%		
Working experience(y)						
< 1	19	3.9%	28	4.72%	16.522	< 0.01
1 ~ 5	157	32.24%	200	33.73%		
6 ~ 10	113	23.2%	188	31.7%		
≥ 10	198	40.66%	177	29.85%		
Marital status					0.486	0.92
Married	343	70.4%	408	68.8%		
Unmarried	135	27.7%	172	29.0%		
Divorced	8	1.6%	12	2.0%		
Other	1	0.2%	1	0.2%		
Having child					3.024	0.082
Yes	331	68.0%	373	62.9%		
No	156	32.0%	220	37.1%		
Employment relationship					12.607	< 0.01
Formal staffing	127	26.1%	105	17.7%		
Contract	357	73.3%	479	80.8%		
Dispatch	3	0.6%	9	1.5%		
Number of night shifts per month					22.965	< 0.01
0 ~ 5	332	68.2%	320	54.0%		
5 ~ 10	128	26.3%	232	39.0%		
≥ 10	27	5.5%	41	6.9%		
Number of patients in day shift nursing					8.880	0.012
1 ~ 8	162	33.3%	151	25.5%		
9 ~ 16	186	38.2%	237	40.0%		
> 16	139	28.5%	205	34.5%		
Number of patients in night shift nursing					2.397	0.302
1 ~ 16	102	20.9%	122	20.4%		
17 ~ 32	112	23.0%	115	19.4%		
> 32	273	56.1%	356	60.0%		

### Inclusive leadership scale scores for obstetrics and gynecology nurses

As shown in Table 3, the average of inclusive leadership score of obstetrics and gynecology nurse was 34.25 ± 7.23, especially in the domains of openness and accessibility, indicating that obstetrics and gynecology nurses had low levels of inclusive leadership.

**Table 3:** Inclusive leadership scores of obstetrics and gynecology nurses (*n* = 1080)

Dimensions	Number of entries (n)	Score range	Average score ($\overline{x }$±s)
Openness	4	4 ~ 20	11.81 ± 2.24
Effectiveness	3	3 ~ 15	13.14 ± 2.46
Accessibility	2	2 ~ 10	11.25 ± 2.15
Total	9	9 ~ 45	34.25 ± 7.23

### Psychological coherence score of obstetrics and gynecology nurses

As presented in Table 4, the average score of psychological coherence score of obstetrics and gynecology nurses was 55.79 ± 8.28, indicating that obstetrics and gynecology nurses had low levels of psychological coherence.

**Table 4:** Psychological coherence score of obstetrics and gynecology nurses (*n* = 1080)

Items	Average score ($\overline{x }$±s)
Sense of understanding	21.13 ± 4.22
Sense of control	19.22 ± 4.14
Sense of meaning	17.01 ± 4.08
Total	55.79 ± 8.28

### Pearson correlation analysis

As shown in Table 5, Pearson correlation analysis showed the openness, effectiveness, accessibility, sense of understanding, sense of control, sense of meaning was all negatively correlated with the implicit absenteeism in obstetrics and gynecology nurses (all *P* < 0.05).

**Table 5:** Pearson correlation coefficient matrix of inclusive leadership, implicit absenteeism, psychological coherence of obstetrics and gynecology nurses (*n* = 1080)

Variables	Implicit absenteeism	Openness	Effectiveness	Accessibility	Sense of understanding	Sense of control	Sense of meaning
Implicit absenteeism	1						
Openness	601^*^	1					
Effectiveness	625^*^	704^*^	1				
Accessibility	613^*^	647^*^	710^*^	1			
Sense of understanding	622^*^	605^*^	638^*^	671^*^	1		
Sense of control	608^*^	623^*^	645^*^	665^*^	684^*^	1	
Sense of meaning	621^*^	633^*^	674^*^	685^*^	704^*^	723^*^	1

^*^P < 0.05

### Mediating effect of psychological coherence between inclusive leadership and implicit absenteeism in obstetrics and gynecology nurses

**Fig. 1:**
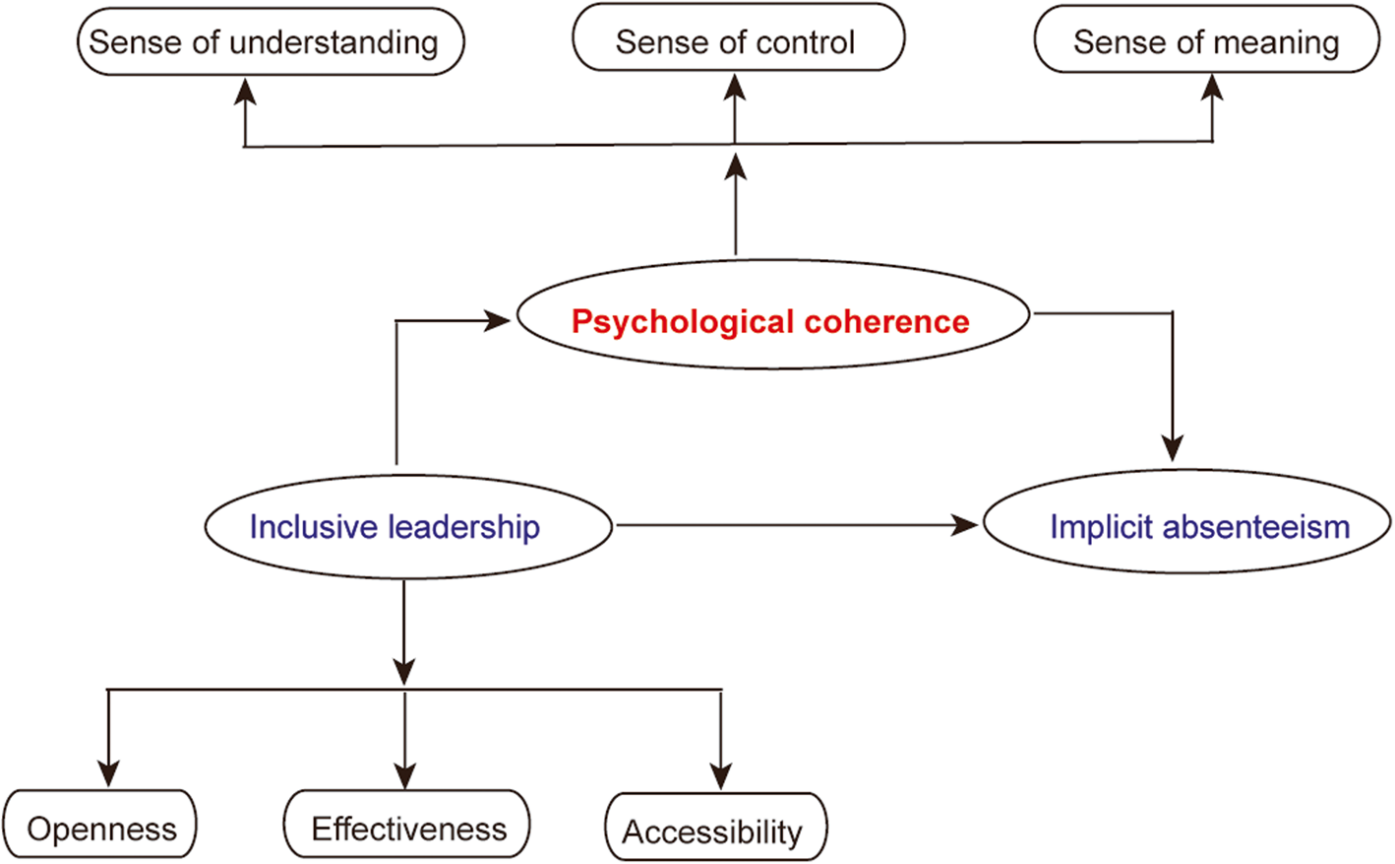
The mediating effect model of psychological coherence between inclusive leadership and implicit absenteeism in obstetrics and gynecology nurses

We conducted regression analysis among dimensions of inclusive leadership, psychological coherence, and invisible absenteeism by building a model with hidden absenteeism as the dependent variable. Model 1: Taking all dimensions of inclusive leadership as independent variables and the sense of psychological coherence as a dependent variable into the model, the results showed that inclusive leadership had a positive effect on the sense of psychological consistency. Model 2: All dimensions of inclusive leadership were used as independent variables, and implicit absence behavior was included in the model as a dependent variable. The results showed that the regression coefficient of inclusive leadership on implicit absence behavior was 0.605, and the F value was significant at the 0.05 level. Model 3: Incorporating the dimensions of inclusive leadership and the sense of psychological coherence as independent variables into the model, and taking implicit absenteeism as the dependent variable, the results showed that the regression coefficient of inclusive leadership on the implicit absence behavior of obstetrics and gynecology nurses was reduced from 0.605 to 0.417, T value and F value test were significantly different, and the regression equation model of psychological coherence and recessive absenteeism behavior was established. Therefore. psychological coherence played a partial mediating role between inclusive leadership and obstetrics and gynecology nurses' implicit absenteeism (Table 6). The model of the mediating effect of psychological consistency in inclusive leadership and obstetrics and gynecology nurses' implicit absence was shown in Fig. 1.

**Table 6:** Analysis of the mediating effect of psychological coherence between inclusive leadership and implicit absenteeism (*n* = 1080)

Steps	Independent variable	Dependent variable	Standard regression coefficients	T	F	△R^2^
1	Inclusive leadership	Psychological coherence	0.581	12.163^*^	50.163^*^	0.286
2	Inclusive leadership	Implicit absenteeism	0.605	12.145^*^	45.062^*^	0.309
3	Inclusive leadership	Implicit absenteeism	0.417	7.056^*^	44.117^*^	0.348
	psychological coherence		0.352	5.267^*^		

^*^P < 0.05

## Discussion

Previous studies [21, 22] have shown that nurses' implicit absenteeism is closely associated with patient falls, more medication errors and lower quality of care. In addition, persistent absenteeism may trigger the deterioration of nurses' own health, leading to more negative impacts such as impaired health productivity and economic loss [23, 24]. Therefore, nurse managers should pay more attention to the needs and pressures of nurses to ensure the quality and safety of nursing through various interventions. In this study, the implicit absenteeism score of obstetrics and gynecology nurses was (21.06 ± 3.22) points, which is higher than that of the research of Ren Wei et al. [25], indicating that obstetrics and gynecology nurses' recessive absenteeism is particularly serious among nurses. Respondents reported that feeling of powerlessness, work pressure and work frustration are common in the work environment. It has been reported [26] that The pressure of the second child in family life in China is the main factor hindering nurses' efficient performance at work. Implicit absenteeism was higher among the sample participants compared with previous reports [27–30]. It may be explained that in China, the professional identity of nurses is relatively low, and the degree of professional recognition is not high [31]. The nurse-patient ratio is relatively low, and the workload of nurses is large and intensive in China [32]. In terms of department management, nurses lack all-round support, causing nurses to feel neglected. On the other hand, the professional identity of nurses is not recognized, which leads to the lack of work enthusiasm of the respondents [33]. It has been reported that at work, nurses are perceived as merely serving staff who obeyed doctor's instructions [34]. Nurses, despite being numerically superior, face challenges in rights, respect, health and comfort in clinical setting [23, 35]. In order to improve the professional status of nurses and create good working conditions for nurses and provide them with all-round support, it is necessary to start from the perspective of management [36, 37]. Inclusive leadership plays an important role in the nurse management [38]. Hospital managers must pay attention to the implicit absenteeism of obstetrics and gynecology nurses, the work pressure and demands of nurses [39]. In this study, the total score of the inclusive leadership scale was (34.25 ± 7.23) points, indicating that the level of inclusive leadership perceived by obstetrics and gynecology nurses was not high. It shows that although the nursing managers of obstetrics and gynecology have a certain openness and amiability in the face of nurses, and they recognize the contributions made by nurses to the department, nurses do not perceive much the leadership.

The results of this study suggest that obstetrics and gynecology nurses have a lower level of psychological coherence. The main reason is that in daily nursing work, obstetrics and gynecology nurses have certain cognition and positive evaluation when facing stressful stimuli, but they still lack certain coping resources to deal with stressful stimuli [40, 41]. At the same time, heavy work, low social support, and unbalanced ratio of nurses to patients will increase the mental and emotional burden of nurses, thereby reducing the level of psychological coherence [42, 43]. Besides, if nurses are exposed to negative environments for a long time, it will also affect their level of psychological coherence [44]. From the perspective of the obstetrics and gynecology department of the hospital at this stage, the patients' demand for nursing is getting higher and higher, and the influence of the patient's independent choice of medical treatment mode has led to aggravation of nurses' work pressure and increased mental pressure [45, 46]. Previous study [47] has found that the psychological coherence of medical staff has a moderating effect on the imbalance of effort and return and health productivity. Therefore, the hospital should pay attention to the improvement of the psychological consistency of the obstetrical nurses.

Previous research by Ding Hui et al. [5] has showed that the level of nurses' psychological coherence is negatively correlated with recessive absenteeism. Previous studies [48, 49] have showed that the frequency of implicit absenteeism decreases with an increase in the level of psychological coherence. In the sense of psychological coherence, the sense of meaning dimension has the strongest correlation with hidden absenteeism. It shows that if nurses do not feel the meaning and value of work, they will treat work negatively [50]. It not only affects the quality of care and work efficiency, but also may cause unpredictable harm to patients, individual and organizations. It is necessary for nursing managers to pay attention to psychological coherence from the perspective of the system, to formulate management systems and intervention measures suitable for the undergraduate room and the hospital, and to provide help for nurses who are under high pressure and lack professional identity [51, 52]. For example, in terms of stress management, we can regularly provide psychological counseling services to nurses through special lectures, psychological counseling, etc. to relieve work pressure. In terms of professional identity, it is possible to help young nurses integrate into the collective through further education, learning, group activities, etc., to strengthen team awareness and professional identity, to enable nurses to find the meaning and value of their work [53, 54]. Institutional and leadership arts can reduce nurses' invisible absenteeism by improving nurses' psychological coherence through incentives and inclusive leadership [55].

The results of this study suggest that psychological coherence plays a partial mediating role between inclusiveness and obstetrics and gynecology nurses' implicit absenteeism behavior. The research of Liang Yulin [56] has pointed out that there is a certain relationship between inclusive leadership and job burnout of medical staff. Therefore, nursing managers should pay attention to the influence of inclusive leadership on the implicit absenteeism of nurses. Develop an inclusive leadership style for nursing managers from the aspects of openness, approachability, and effectiveness [57]. In daily nursing work, nursing managers should fully respect nurses and be good at listening to nurses' ideas, so that nurses can gain a sense of respect and recognition in their leadership, and then actively participate in clinical nursing work [58]. Furthermore, inclusive leadership is a bottom-up leadership style [59]. It pays attention to the affirmation of nurses' abilities and contributions, so that nurses can perceive the support from the department. The stronger this perception, the higher their enthusiasm for work. It is helpful to establish a management example in the whole hospital, promote the inclusive leadership style, and create a good management relationship [60, 61]. Therefore, when cultivating inclusive leadership, it is necessary to strengthen the improvement of nurses' psychological coherence [62]. Through psychological training, strengthening communication and listening, the level of psychological consistency of obstetrics and gynecology nurses can be improved, and nurses can be encouraged to carry out effective self-management and strengthen their own positive emotions, so as to reduce the impact of health problems on work status, and thus improve nursing quality.

Several limitations in this present study must be considered. We could not include the confounding factors in the demographic differences for further analysis because the sample size was small in every specific item. Currently, there are few studies on the mediating effect of inclusive leadership and implicit absenteeism. This study only selected nurses from the obstetrics and gynecology department in Anhui Province as the research population, and there are certain limitations in sample selection, which needs to be further explored in future larger-sample studies.

## Conclusions

To sum up, the nurses’ implicit absenteeism is serious, and the level of psychological coherence is low, and the perception level of inclusive leadership style is not high. Psychological coherence has a certain mediating effect between inclusive leadership and obstetrics and gynecology nurses' implicit absenteeism. By improving a sense of psychological coherence and promoting an inclusive leadership style, it may help reduce the incidence of implicit absenteeism. Furthermore, it is necessary to relieve nurse’s pressure and improve their professional identity.

## Data Availability

All data generated or analyzed during this study are included in this published article.
