# Correction: Psychological coherence, inclusive leadership and implicit absenteeism in obstetrics and gynecology nurses: a multi-site survey

**DOI:** 10.1186/s12888-022-04436-7

**Published:** 2022-12-30

**Authors:** Yu Jin, Qingquan Bi, Guiqi Song, Jun Wu, Hui Ding

**Affiliations:** 1 Binhu Healthcare Center, The First Affiliated Hospital of USTC, Division of Life Sciences and Medicine, University of Science and Technology of China, Hefei, Anhui 230001 China; 2 Department of Geriatrics, The First Affiliated Hospital of USTC, Division of Life Sciences and Medicine, University of Science and Technology of China, Hefei, Anhui 230001 China; 3 Department of Education, The First Affiliated Hospital of USTC, Division of Life Sciences and Medicine, University of Science and Technology of China, Hefei, Anhui 230001 China

**Correction: BMC Psychiatry 22, 525 (2022)**

**https://doi.org/10.1186/s12888-022-04137-1**

Following the publication of the original article [1], the authors would like to remove affiliation 1.

1Hefei, China.

The original article [1] has been corrected.

## References

[1] Jin (2022). Psychological coherence, inclusive leadership and implicit absenteeism in obstetrics and gynecology nurses: a multi-site survey. BMC Psychiatry.

